# Agriculture intensifies soil moisture decline in Northern China

**DOI:** 10.1038/srep11261

**Published:** 2015-07-09

**Authors:** Yaling Liu, Zhihua Pan, Qianlai Zhuang, Diego G. Miralles, Adriaan J. Teuling, Tonglin Zhang, Pingli An, Zhiqiang Dong, Jingting Zhang, Di He, Liwei Wang, Xuebiao Pan, Wei Bai, Dev Niyogi

**Affiliations:** 1 College of Resources & Environmental Sciences, China Agricultural University, Beijing, 100193, China; 2 Department of Earth, Atmospheric, and Planetary Sciences, Purdue University, West Lafayette, Indiana, 47907, USA; 3 Pacific Northwest National Laboratory, Joint Global Change Research Institute, College Park, Maryland, 20740, USA; 4 Department of Agronomy, Purdue University, West Lafayette, Indiana, 47907, USA; 5 Department of Earth Sciences, VU University Amsterdam, Amsterdam 1081 HV, The Netherlands; 6 Laboratory of Hydrology and Water Management, Ghent University, B-9000 Ghent, Belgium; 7 Hydrology and Quantitative Water Management Group, Wageningen University, 6708PB Wageningen, The Netherlands; 8 Department of Statistics, Purdue University, West Lafayette, Indiana, 47907, USA; 9 China Center for Urban Development, Beijing, 100045, China

## Abstract

Northern China is one of the most densely populated regions in the world. Agricultural activities have intensified since the 1980s to provide food security to the country. However, this intensification has likely contributed to an increasing scarcity in water resources, which may in turn be endangering food security. Based on *in-situ* measurements of soil moisture collected in agricultural plots during 1983–2012, we find that topsoil (0–50 cm) volumetric water content during the growing season has declined significantly (*p* < 0.01), with a trend of −0.011 to −0.015 m^3^ m^−3^ per decade. Observed discharge declines for the three large river basins are consistent with the effects of agricultural intensification, although other factors (e.g. dam constructions) likely have contributed to these trends. Practices like fertilizer application have favoured biomass growth and increased transpiration rates, thus reducing available soil water. In addition, the rapid proliferation of water-expensive crops (e.g., maize) and the expansion of the area dedicated to food production have also contributed to soil drying. Adoption of alternative agricultural practices that can meet the immediate food demand without compromising future water resources seem critical for the sustainability of the food production system.

Droughts are one of the most costly natural hazards in China due to their unusual severity and persistence^1^; they resulted in an average annual loss of $37.9 billion during 1978–2003^2^. While recent studies indicate that globally droughts are not intensifying on average^3^, Northern China (NC) is currently facing increasing water scarcity^1^,^4^,^5^. In fact, China is one of thirteen countries considered to be facing extreme water shortages, as the water resources per capita is only one quarter of the world average^6^. Additionally, the distribution of water resources in China does not correspond well with the geospatial distribution of water demands. With only 18% of China’s water resources, NC accounts for as much as 65% of the arable land^6^ and 40% of the population^7^. This imbalance leads to a high exploitation of water in NC, which induces severe ecological and environmental problems derived from the degradation of land and freshwater^8^. In the past three decades, droughts affected an average area of 23.9 million ha per year, and 35.6% of agricultural area in NC^9^. Moreover, recent studies indicate that dry extremes are prone to become more frequent and intense in this region^10^,^11^.

While the soil dry-out in NC has been well documented^1^,^4^,^5^, the drivers of this dry-out and the role of agricultural intensification remain understudied. Changes in soil water content are affected by a large number of factors, such as soil properties (e.g., soil texture, structure, organic matter, depth, density and salinity), climate (e.g., precipitation, solar radiation, temperature, etc.), topography and land cover. These factors regulate infiltration, permeability, water holding capacity and moisture loss rates^12^. Current agricultural practices, such as crop type selection, agronomic measures, fertilizer application, or irrigation management, are expected to change the water use and the dynamics of soil moisture^13^ due to their impacts on the physical and biogeochemical interactions within ecosystems^14^,^15^. Despite recent analyses of meteorological droughts focusing on trends in rainfall^3^,^4^,^5^,^6^,^10^, and studies of model-simulated or satellite-retrieved soil moisture changes^1^,^16^, there is a lack of ground-truth based observational assessments of soil moisture trends. Moreover, the regional impacts of the intensification of agricultural practices remain poorly understood. Consequently, investigating the mechanisms behind the NC dry-out remains crucial for the improvement of regional agricultural and water resource management, and it can provide useful insights that are transferable to other regions experiencing similar water-stress situations^14^,^17^.

This study aims at understanding soil moisture trends in NC using robust observational evidence, and unravelling the impacts of regional agricultural practices on these trends. We first use satellite remote sensing, long-term discharge measurements and meteorological observations to assess the regional drying during the past three decades. Then we use unique long-term *in-situ* soil moisture records to evaluate observed soil drying in agricultural plots. The effects of agricultural intensification on soil moisture decline are assessed through multiple linear regression analysis using data for fertilizer usage and information on crop types and crop area. The Xinjiang autonomous region and the Qinghai province are excluded from the analysis, as population density is low^7^, agriculture is not extensive^18^, and there is a lack of historical data.

## Results

**Figure 1:**
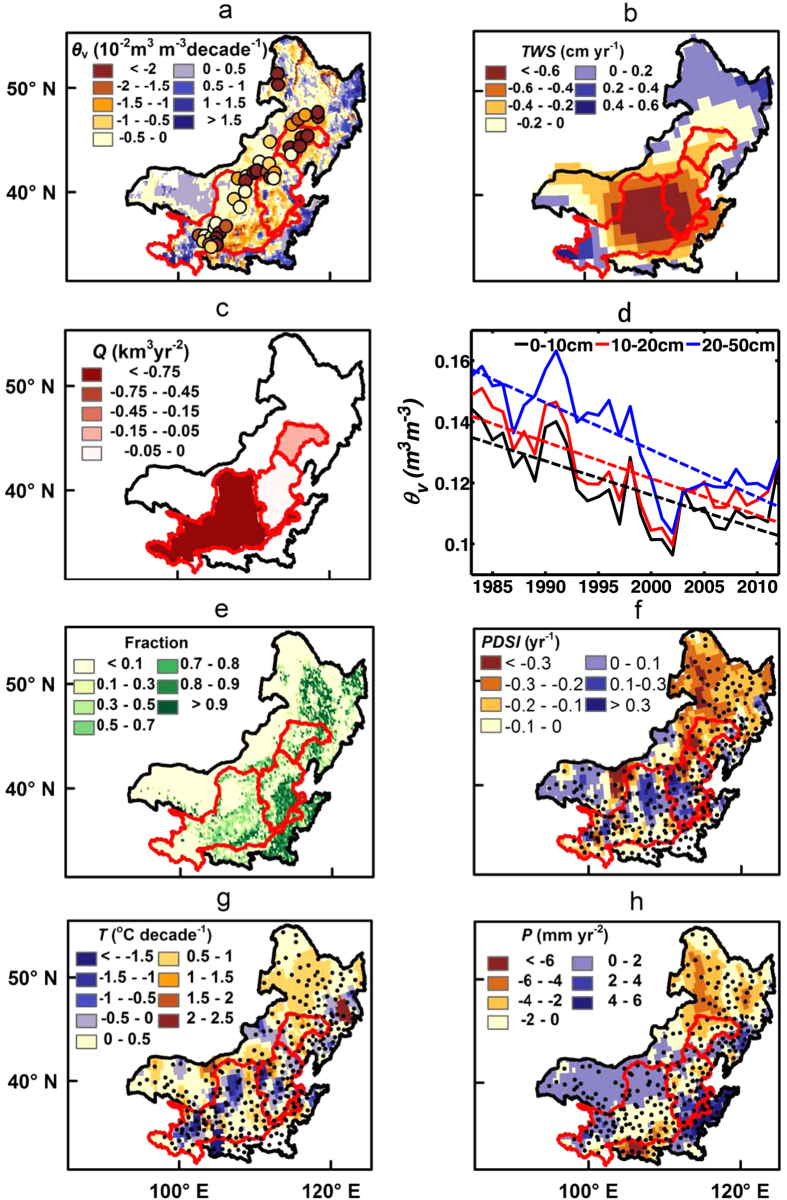
Growing season trends in Northern China (NC) : **a**) Volumetric soil moisture (*θ*_v_) trend during 1983–2010 across NC and at the 40 agricultural meteorological stations (circles on the map) during 1983–2012. The former is derived from CCI-WACMOS soil moisture product^16^ and the latter is from *in-situ* measurements of the top 0–10 cm soil. The red polygons delineate the boundaries of the Yellow, Haihe and Liaohe river basins from west to east, respectively. Note that the boundary of Liaohe river basin is only extended to Tieling gauge station. **b**) Terrestrial water storage (*TWS*) trend during 2003–2013. **c**) Discharge (*Q*) trends in the three basins during 1980–2012. **d**) Average *θ*_v_ trends in 0–10 cm, 10–20 cm and 20–50 cm soil across the 40 agricultural meteorological stations during 1983–2012. **e**) Fraction coverage of cropland in 2010. **f**–**h**) Palmer Drought Severity Index (*PDSI*), air temperature (*T*) and Precipitation (*P*) trend during 1984–2007. The black dots in **f**–**h**) represent the locations of 307 weather stations. All maps are generated via ArcMap10.2, and their projected coordinate systems are Asia North Albers Equal Area Conical.

Satellite observations of surface soil moisture and total terrestrial water storage both point to NC as a hot-spot region of declining water availability at the global scale^16^,^19^ (Fig. 1a,b). Moreover, the growing season river discharges (*Q*) at the outlets of the Yellow, Haihe and Liaohe river basins all show significant negative trends during 1983–2012 (Fig. 1c). This is consistent with the aforementioned satellite-observed regional drying, although dam construction, water withdrawal and groundwater abstraction have also altered the regional water distribution^20^. The Yellow river basin exhibits the largest decline, which is consistent with the reports of the frequent dry-out of the lower reach since 1972^21^.

*In situ* soil moisture measurements across NC reveal a significant soil moisture decline at dryland crop sites. At the 40 agricultural meteorological stations in NC (Fig. 1a), soil moisture was monitored 3–5 times per month using the gravimetric technique during the growing season (generally April-October). These stations cover rain-fed croplands where a variety of different crop types were grown. Across these 40 stations, we find that the average volumetric soil moisture (*θ*_v_) during growing season decreased significantly in the last three decades (Fig. 1d). We find a significant (*p* < 0.01) *θ*_v_ trend of −0.011, −0.012 and −0.015 m^3^ m^−3^ per decade in the top 0–10 cm, 10–20 cm, and 20–50 cm of soil (i.e., the fitted *θ*_v_ in 20–50 cm of soil decreased from 0.157 m^3^ m^−3^ in 1983 to 0.112 m^3^ m^−3^ in 2012 or by about 29%, see Fig. 1d), respectively. Similar negative trends (−6.2% to −10.4% per decade) are also found in the soil moisture index (SMI, Fig. S1), a measure of soil moisture content as ratio of the total potential storage available to plants^22^. This analysis thus provides evidence for a notable soil moisture decline at agricultural fields across NC.

The trends in satellite-observed surface soil moisture are generally consistent with the ground-measured top 10 cm soil moisture at the 40 stations (Fig. 1a, correlation coefficient *r* = 0.75). Disagreements in the magnitude of these trends might occur due to the scale-mismatch between the point measurements and the satellite footprint, and also because crops do not necessarily cover the entire area of each pixel^18^ (Fig. 1e). Additionally, the satellite sensors usually detect the conditions in the top 0.5–2 cm of soil, rather than the 0–10 cm of soil sampled by the *in-situ* measurements.

The detected soil moisture decline over the last three decades could have multiple drivers, including climate change. In fact, an overall decline of the Palmer Drought Severity Index^23^ (PDSI, Fig. 1f) – a widely used metric to monitor meteorological droughts – reflects the simultaneous spatiotemporal increase in air temperature (Fig. 1g, Fig. S2a) and decrease in rainfall (Fig. 1h, Fig S2b) in the region over this period. The most dramatic decline in PDSI occurs in the northeast part of Inner Mongolia, where a pronounced warming and drying trend is also found. Trends in other drought indices such as Standardized Precipitation Index (SPI)^24^ and Standardized Precipitation Evapotranspiration Index (SPEI)^25^ further support these results (Fig. S3). The negative trends for SPEI are more prominent than that for SPI, suggesting that the increased atmospheric demand for water has played a role as well^25^. These results suggest that climate change has contributed to soil drying in NC^26^.

We disentangle the effects of climate change from those of agricultural practices using a multiple linear regression analysis. Note that soil properties, microclimate and topography intrinsically affect soil moisture content^12^ and may vary from county to county due to heterogeneity in the environmental conditions; their effects on soil water content may overshadow the effects induced by agricultural practices^12^,^27^. We define the variable “county effect” (*C*_*m*_) to account for differences in soil moisture level caused by differences in the environmental conditions across different counties/stations. Other than *C*_*m*_, results show that among all the meteorological and agricultural explanatory variables considered (see Methods), precipitation (*P*), fertilizer use rate (*F*) and crop type (*c*) are most significant in influencing the soil moisture at the three soil depths considered. Here we take the 20–50 cm soil layer, which comprises a large portion of root zone^28^,^29^ and is directly affected by transpiration, to illustrate the correlated effects of declining soil moisture. The analysis yields slopes of 4.1 × 10^−4^ (m^3^ m^−3^ mm^−1^ yr), −3.0 × 10^−4^ (m^3^ m^−3^ kg^−1^ ha yr), −0.013 and −0.026, for *P*, *F*, crop group 2 (i.e., *c*2, wheat) and group 3 (i.e., *c*3, maize and rapeseed), respectively – see model (1) in Methods. So, for instance, while a 100 mm increase in growing season precipitation could lead to an increase of 0.041 m^3^ m^−3^ in *θ*_v_, a 100 kg ha^−1^ increase in fertilizer use could lead to an 0.030 m^3^ m^−3^ reduction in *θ*_v_ over 1983–2006, which is approximately 20% of the mean observed *θ*_v_ (Fig. 1d).

The model explains 81% of variation in *θ*_v_, indicating that the changes in soil moisture can be reasonably well captured by the explanatory variables considered. A large fraction (60%) of the total variation explained by the model (81%) is due to *C*_*m*_; however, the average variation derived from *C*_*m*_ adjusted by the degree of freedom (*df* *=* *39*) is small. In terms of the relative contribution, *P*, *F* and *c* account for 32%, 38% and 30%, respectively, of the the remaining model variation (40% of the 81% explained by the model). The 32% relative contribution of *P* indicates that climate change induced soil moisture decline is substantial. We do not find *T* has significant impact on *θ*_v_. This suggests that *θ*_v_ is more sensitive to *P*, and that the impact of water supply overshadows that of atmospheric evaporative demand increase induced by warming.

**Figure 2:**
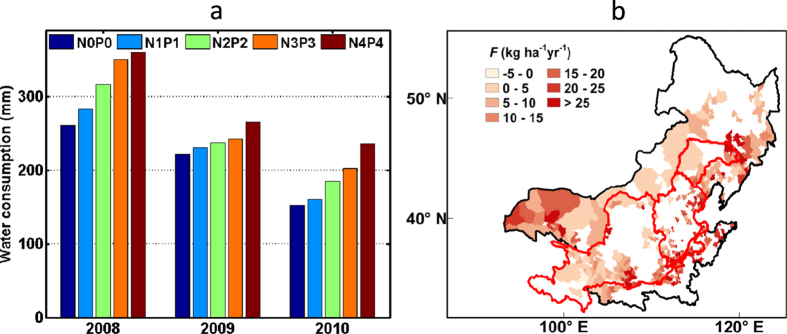
Fertilizer use experiment and fertilizer use trend in Northern China : **a**) water consumption in N0P0-N4P4 fertilizer use treatments between 2008–2010, which is derived from fertilizer use experiment in Wuchuan Field Experiment Station, where N0P0–N4P4 stand for increasing fertilizer use treatments that correspond to Table S1; and **b**) fertilizer use (*F*) trend during 1983–2006 across the region. Note that area without fertilizer use data is left vacant. The map in **b**) is generated via ArcMap10.2, and the projected coordinate system is Asia North Albers Equal Area Conical.

**Figure 3:**
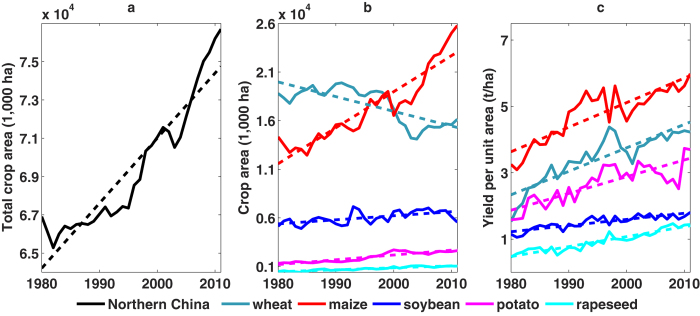
Changes in crop area and yield per unit area in Northern China during 1980–2011 : **a**) total crop area in the region; **b**) planting area for each major crop; and **c**) yield per unit area for each major crop.

The positive covariance between *P* and *θ*_v_ is expected, as *θ*_v_ responds directly to changes in *P*. However, the considerable contribution of *F* and *c* to *θ*_v_ decline is of interest and may be less straightforward. In order to further explore this negative correlation, we examine data gathered at the Wuchuan Field Experiment Station, where the relations between soil moisture and fertilizer loads were monitored from 2008–2010. In this experiment, the effect of fertilizer was monitored in isolation as fertilizer use was the only changing agricultural variable, and other agricultural practices such as tilling method, plant variety and plant density were the same across different treatments and along time. Results show a clear rise in field water consumption with increased fertilizer use, by 4%–54% depending on the specific fertilizer treatment (Table S1, S2, Fig. 2a). In addition, the choice of crop types also leads to significant differences in terms of *θ*_v_. Taking the crop group 1 (i.e., *c*1, soybean and potato) as baseline, regression results indicate that the choice of crop group *c*2, and especially *c*3, has negative effects due to their higher water consumption. This is consistent with the findings from previous field experiments conducted in NC^30^,^31^,^32^,^33^,^34^,^35^ that indicate that crop water consumption during the growing season follows the order: *c*1 (326–452 mm) < *c*2 (450–500 mm) < *c*3 (398–568 mm). These results suggest that the increased fertilizer use (Fig. 2b) and the associated agricultural practices such as crop area expansion (Fig. 3a) in NC^9^,^18^– with a disproportionate proliferation of high water-consuming crops (e.g., maize, Fig. 3b) – have contributed to the widespread soil moisture decline in the region.

The factors that impact discharge decline in NC are also assessed for the three river basins based on multiple linear regression models. Results are similar for each of the three basins. In the following, we focus on the Yellow river basin, due to its larger coverage and dense population. Our regression model for the Yellow river (see model (2) in Methods section) explains 82% of the long-term variation in discharge. *P* has a significant positive effect on *Q* (slope of 0.12 km^3^ mm^−1^), whereas *F* and *A*_w_ (area covered by wheat plantations) have significant negative effects (−0.29 km^3^ kg^−1^ ha and −0.06 km^3^ 10^−3^ ha^−1^, respectively). The crop area of maize (*A*_mz_) is not considered to have significant effects on *Q* (i.e., *p* = 0.97) despite the high correlation between *A*_mz_ and *Q* (*r* = −0.76, *p* < 10^−5^). This occurs due to the “Simpson Paradox”^36^, in which two strongly dependent variables like *F* and *A*_mz_ (*r* = 0.92) cannot co-exist in the model. Replacing *A*_w_ with *A*_mz_ in the model, we find *A*_mz_ also has significant negative effect on *Q* (*p* < 0.001). Overall, the effects of *P*, *F*, acreage of maize and wheat on *Q* are consistent with those on *θ*_v_. This finding suggests that extensive agriculture intensification in the Yellow, Haihe and Liaohe river basins – where 26.6%, 48.2% and 34.1% of the area is covered by cropland in 2010^18^ – could partly explain the *Q* declines.

**Figure 4:**
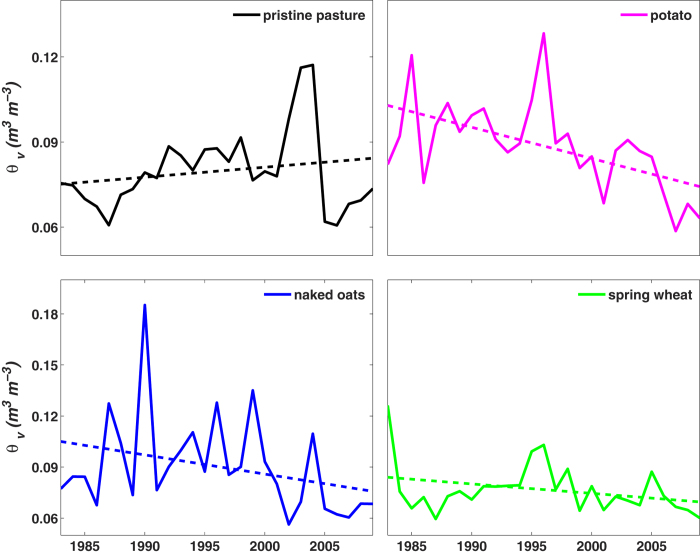
Variation of volumetric soil moisture (*θ*_v_) in topsoil (0–50 cm) of pristine pasture and different crop fields in Wuchuan Agricultural Meteorology Observation Station during 1983–2009.

A pair-wise experiment conducted at the Wuchuan Agricultural Meteorology Observation Station further supports the hypothesis that agricultural intensification accelerates soil moisture decline. For nearly three decades (1983–2009), soil moisture has been monitored at two contiguous sites: a pristine pasture and an agricultural site. Measurements reveal a significant (*p* < 0.05) negative trend of soil moisture in the first 50 cm of soil for the cropland (for all crop types), contrasting with the slightly positive – but statistically significant (*p* < 0.05) – trend observed in the pristine pasture (Fig. 4). In potato, naked oats and spring wheat fields, *θ*_v_ decreased by 0.011, 0.011 and 0.006 m^3^ m^−3^ per decade, respectively. For instance, the fitted *θ*_v_ decreased from 0.103 m^3^ m^−3^ in year 1983 to 0.074 m^3^ m^−3^ in 2009 at the potato field. However, in the pristine pasture – where trends reflect larger scale climatic change only – there was an increase of 0.003 m^3^ m^−3^ per decade. Since no significant differences in soil texture, topography and microclimate exist between the cropland and pasture sites, differences in *θ*_v_ may be attributed to the agricultural activities.

## Discussion

Food sufficiency has been achieved after nearly 50 years of sustained efforts in China^37^. Since the early 1980s, agriculture has intensified dramatically, and the food production system currently relies on application of chemical fertilizers and implementation of modern farming techniques^38^. The amount of fertilizer use in China accounts for 31.4% of the total global consumption^39^; this can be considered as a very inefficient system^40^, with rates of fertilizer application that are far greater than the needs of crops^37^,^41^,^42^,^43^. While the yield per hectare for all crops has increased from 1.21 t ha^−1^ to 4.83 t ha^−1^, the yield per unit chemical fertilizer use has decreased dramatically for the three main crops in China during 1961–1998: 164 to 10 kg kg^−1^ for rice, 44 to 6 kg kg^−1^ for wheat and 93 to 9 kg kg^−1^ for maize^44^.

Although the effects of excessive fertilizer use on agriculture and environment in China have been investigated in recent years^37^,^42^,^43^,^45^, only a few studies have drawn the attention to the potential effects of fertilizers on transpiration rates, thus on the hydrological cycle^46^,^47^. Excessive fertilizer use can increase water consumption and deplete soil moisture as revealed by our results (Fig 2a), while having little impact on crop yield^37^. In addition, fertilizer use and other agricultural practices may aggravate soil salinity and soil compaction, leading to decrease of infiltration rates and soil water holding capacity^12^, which further limits the available soil water.

It is important to note that limitations of this study arise from the scarcity of data on regional agricultural practices and the inability to capture all the interactive factors involved within a statistical framework. The increase in fertilizer use and the corresponding decline in soil moisture should be interpreted more broadly; changes in fertilizer use may be accompanied by the incorporation of new plant varieties, modified phenotypes or changes in agricultural technology, and therefore reflect a broad scenario of agricultural intensification. The increase in the yield per unit area for each of the main crops (wheat, maize, soybean, potato and rapeseed, Fig. 3c) is also an integrated effect of wide range of agricultural practices. New crop varieties have been extensively introduced in NC to enhance yields and improve crop quality in recent years^48^. These new varieties have different water and fertilizer use^48^, and thus potentially affect soil moisture. Similarly, irrigation area has drastically expanded^20^,^49^ and a range of farming techniques (e.g., reduced tillage, mulching) have been widely implemented in recent years across NC^50^. However, lack of historical information about cultivation of the large number of varieties – often exceeding 10,000 for individual crops^48^, irrigation management and implementation of farming techniques across different areas and over different eras make it infeasible to extract their impacts on regional soil moisture at present. Therefore, the results on fertilizer use reflect only the overall intensification of agriculture, whereas local factors that might have contributed to this intensification and to the decline in soil moisture (e.g. pesticide and herbicide application, changes in crop varieties, improvement in on-farm practices and implementation of modern technologies) could not be investigated at this stage.

Water scarcity has become increasingly prominent in NC as agriculture has intensified^51^,^52^. Current agricultural practices paired with inappropriate nutrient and irrigation management lead to severe environmental degradation^43^,^45^ and excessive exploitation of water resources^50^, as well as low nutrient use efficiency and water use efficiency that lag behind global averages^51^. As a result, alternative agriculture practices that could help meet food demand without compromising future water resources need to be pursued for sustainable agriculture. Recent years have seen progress in research on agriculture management^8^,^37^,^50^,^51^,^52^, contributing to addressing the conflict between aggravating resources shortage and increasing food demand. The implementation of integrated soil-crop system management (e.g. synchronization of nutrient supply and crop demand)^8^,^52^, the dissemination of water-saving technologies (e.g., mulching, drip irrigation), agronomic measures (e.g., reduced till, recycling of organic manures) and agro-climatic decision tools (using crop-climatic relations or data analytics), and the enhancement in crop varieties with resistance to drought and heat seem critical for sustainable agriculture production in NC.

## Methods

We analyse the soil moisture trends over NC using long-term measurements from 40 agricultural meteorological stations during 1983–2012. We support this analysis with a study of the discharge (*Q*) trends from the three outflow river basins in the region. To isolate the long-term impact of precipitation trends on the soil moisture changes, we examine how meteorological drought has evolved across NC using meteorological variables such as precipitation and air temperature from 307 weather stations. We use a Mann-Kendall test to evaluate the significance of the trends. Multiple linear regression is used to assess the correlated effects for changes in soil moisture and *Q*. Details of observed data and ancillary data used in this study are provided in the Supplementary Information.

Since the locations of the 40 stations, where the soil moisture measurements are conducted, are different from those of the weather stations, the meteorological data used for each site in the regression is interpolated from observations at four nearest weather stations using the inverse distance weighting method^53^. Historical records of (chemical) fertilizer use at these 40 stations are not available. To cope with this site-level data scarcity we use county-average rates^54^. Data at these 40 stations is combined into one multiple regression model.

In the regression for the soil moisture changes, we consider the impacts of meteorological variables such as air temperature (*T*, 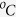), precipitation (*P*, mm yr^−1^), wind speed (*u*, m s^−1^), relative humidity (*RH*) and radiation hours (*R*, hr), and agricultural activities as reflected by fertilizer use rate (*F*, kg ha^−1^ yr^−1^, calculated as total weight of fertilizer use divided by cropland area) and crop types (*c*), as well as “county effect” representing differences in soil moisture level across different counties (*C*_*m*_, the overall effects of county level environmental variables such as topography, soil texture and microclimate). We jointly consider nitrogen, phosphorus, and potassium fertilizers due to the unavailability of historical records of their separate use. These fertilizers we considered are active ingredients as nitrogen, phosphorus pentoxide, and potassium oxide, respectively. Based on multiple experiments on the crop water consumption that have been conducted in NC region^30^,^31^,^32^,^33^,^34^,^35^, we categorize the main crop types in NC into three groups: (i) soybean and potato (*c*1); (ii) wheat (*c*2); and (iii) maize and rapeseed (*c*3).

We use the ANCOVA model^55^ to investigate the correlated effects between the dependent and explanatory variables. The model can be summarized as:


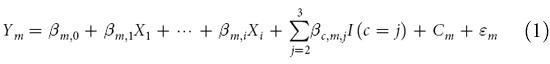


where 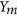 is the mean growing season *θ*_v_ for each year during 1983–2010. 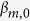 is the intercept term, 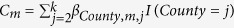 represents the county effect, X_1_ ~ X_i_ (i.e., *T*, *P*, *RH*, *R*, *u* and *F*) represent the *ith* explanatory variable and 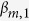 ~ 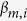 are the corresponding slopes, 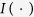 is the indicator function, and 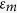 is a normally distributed error term. Since we have classified the crops into three groups, we use two parameters (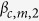 for group 2 and 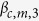 for group 3) to represent the effects of crop groups. The factor terms do not contain the first level in their expression since we choose the first level of both county effect and crop effects as baselines. Note that the choice of baseline does not affect the regression results. Analysis of variance method^56^ is used to quantify the contribution of each variable to the total variation in the model.

We build separate models for the three river basins to investigate the effects of meteorological and agricultural variables on changes in *Q*. We use discharge data at the outlets of each river basin as dependent variable. Explanatory variables involve meteorology (i.e., *T*, *P*, *RH*, *R* and *u*) and agricultural activities (i.e., *F*, crop area of each major crop type) aggregated from all counties within each basin. The full model with all explanatory variables is given by:


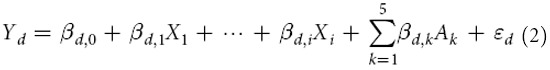


where 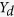 stands for the growing season mean *Q* for each year during 1983–2012 (km^3^ yr^−1^), 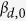 is the intercept term, X_1_~X_i_ (i.e., *T*, *P*, *RH*, *R*, *u* and *F*) represent the *ith* explanatory variable and 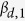 ~ 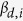
are corresponding slopes, 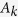
stands for the planting area of *kth* crop (i.e., wheat, maize, soybean, potato, rapeseed) and 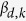 is the corresponding slope, and 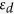 is a normally distributed error term. Besides this full model, a few reduced models that assign some of the coefficients equal to zero are also investigated. This is important because of the possible impact of the “Simpson Paradox” on parameter estimates as well as on the significance of effects^36^.

When we calculate the Palmer Drought Severity Index (*PDSI*)^23^ and the Standardized Precipitation Evapotranspiration Index (SPEI)^25^ we use the FAO56 Penman-Monteith equation^57^ instead of the Thornthwaite equation to estimate potential evapotranspiration (PET)^3^.

## Additional Information

**How to cite this article**: Liu, Y. *et al.* Agriculture intensifies soil moisture decline in Northern China. *Sci. Rep.* **5**, 11261; doi: 10.1038/srep11261 (2015).

## Supplementary Material

Supplementary Information
