# Supplementary material for: Agriculture intensifies soil moisture decline in Northern China
Source: Sci Rep. 2015 Jul 9;5:11261. doi: 10.1038/srep11261 (PMC4497304; doi:10.1038/srep11261)
Supplement: Supplementary Information [file srep11261-s1.pdf]

Supplementary Information

Article title: Agriculture intensifies soil moisture decline in Northern China

Journal name: *Scientific Reports*

Author names: Yaling Liu, Zhihua Pan, Qianlai Zhuang, Diego G. Miralles, Adriaan J. Teuling, Tonglin Zhang, Pingli An, Zhiqiang Dong, Jingting Zhang, Di He, Liwei Wang, Xuebiao Pan, Wei Bai, Dev Niyogi

Affiliations and emails of Corresponding authors: College of Resources & Environmental Sciences, China Agricultural University, Beijing, 100193, China，panzhihua@cau.edu.cn; Department of Earth, Atmospheric, and Planetary Sciences, Purdue University, West Lafayette, Indiana, 47907, USA, qzhuang@purdue.edu.

The supplementary information includes detailed description of data used in this study (Section 1), and fertilizer use experiment (Section 2).

1 Data

The observations used in the analyses include: (a) soil moisture measurements during growing season (generally April-September, 3-5 observations per month) from 40 different agricultural meteorological stations across Northern China for 1983–2012 (Fig. 1a, note that these stations are located in rain-fed dry-land area and no irrigation is applied), and the soil moisture is measured using the gravimetric technique; (b) a long-term monitoring-field data set of soil moisture in pristine pasture and different crop fields in the Wuchuan Agricultural Meteorology Observation Station for 1983-2009; (c) a dataset on crop water consumption with different fertilization use in the Wuchuan Field Experiment Station in Inner Mongolia of China for 2008-2010; (d) monthly discharge data at the outlets of the three outflow basins (obtained from Ministry of Water Resources of China), Yellow, Haihe and Liaohe river basin for 1980-2012, note that we use the discharge data at Tieling gauge station, which is close to the outlet, for the Liaohe river basin as there is frequent seawater backwash at the outlet; (e) meteorological observations from 307 weather stations across Northern China (obtained from Chinese Meteorological Administration), which include monthly air temperature, precipitation, wind speed, relative humidity and radiation hours. We then use the methods in Andrews (2010) to derive vapour pressure deficit from air temperature and relative humidity[1]. These meteorological observations are

interpolated into 0.5°×0.5° spatially explicit regional meteorological data via inverse distance weighting method[2] for regional analysis.

Historical records contain county level and province level agricultural statistical data for 1983-2010 (obtained from National Bureau of Statistics of China), which include the crop area and yield for each major crop, total fertilizer use weight, total crop area. Note that the sequence of administrative hierarchy in China is province, city, county, town and village.

Other ancillary data include satellite-based soil moisture, land-use/land-cover change (LUCC) and radiation products. Satellite retrieved CCI-WACMOS land surface soil moisture data[3] that merges active and passive microwave observations and refers to a depth of 0.5-2cm during 1983-2010, and GRACE terrestrial water storage data – the mean of three products CSR, GFZ and JPL ensemble[4] – during 2003-2013 are also used in this study to investigate the trend in soil water availability and understand their magnitude within the global context. Land-use/land-cover data of year 2010 derived from Landsat TM/ETM digital images[5] are used to calculate fraction coverage of cropland. Monthly average NASA/GEWEX Surface Radiation Budget (SRB) Release-3.0 data sets during 1984-2007 are used to calculate potential evaporation via FAO-56 Penman-Monteith equation[6], and subsequently the Palmer Drought Severity Index[7] and the Standardized Precipitation Evapotranspiration Index[8].

2 Fertilizer use experiment

The impact of different fertilizer use on water consumption of potato was conducted at Wuchuan Field Experiment Station (41.60°N，111.27°E) in Inner Mongolia of China from 2008 to 2010. This station is located in the middle of the north agro-pastoral transitional zone, and is considered representative of semi-arid farming-pastoral transitional area[8]. Experiment was designed as an increasing gradient of fertilizer use rate: N0P0, N1P1, N2P2, N3P3, and N4P4 (Table S1). Fertilizer was applied before planting in each plot. Each plot area was 6 × 10 m^2, and the row spacing was 50 cm, and the planting density was 6 m^{-2}. Soil moisture was measured 3-5 times per month using gravimetric technique, and the water consumption for the whole growing season was then calculated using the water balance method[9], and the runoff was neglected as the station is located in semi-arid dry-land agriculture region.

References

1. Andrews D. G. *An introduction to atmospheric physics*. (New York, Cambridge University Press, 2010).
2. Shepard, D. *Two-dimensional interpolation function for irregularly-spaced data*: 23rd ACM national conference, Las Vegas, Nevada, USA. New York: Association for Computing Machinery Press, doi: 10.1145/800186.810616 (1968, August 27-29).
3. Dorigo W. J. R., *et al*. Evaluating global trends (1988 - 2010) in harmonized multi - satellite surface soil moisture. *Geophys Res Lett* **39,** doi:10.1029/2012GL052988 (2012).
4. Sakumura C., Bettadpur S. & Bruinsma S. Ensemble prediction and intercomparison analysis of GRACE time variable gravity field models. *Geophys Res Lett* **41,** 1389-1397 (2014).
5. Liu J., *et al*. The land use and land cover change database and its relative studies in China. *J Geographical Sciences* **12,** 275-282 (2002).
6. Allen R. G., Pereira L. S., Raes D. & Smith M. *Crop evapotranspiration-Guidelines for computing crop water requirements-FAO Irrigation and drainage paper 56*. (Rome, FAO, 1998).
7. Palmer W. C. *Meteorological drought*. (US Department of Commerce, Weather Bureau Washington, DC, USA 1965).
8. Vicente-Serrano, S. M., Beguería, S. & López-Moreno, J. I. A multiscalar drought index sensitive to global warming: the standardized precipitation evapotranspiration index. *J Climate* **23**, 1696-1718 (2010).
9. Zhao L., *et al*. Impacts of recent climate change on dry-land crop water consumption in the northern agro-pastoral transitional zone of China. *Acta Meteorologica Sinica* **27**, 585-590 (2013).

Table S1 Fertilizer use experiment design

**number**	**treatment**	**Pure fertilizing amount（kg ha^{-1}）**		**Actual fertilizer amount（kg ha^{-1}）**	
		N	P_2O_5	Urea	Triple superphosphate
1	N0P0	0	0	0	0
2	N1P1	45	45	97.5	97.5
3	N2P2	90	90	195	195
4	N3P3	135	135	294	294
5	N4P4	180	180	391.5	391.5

Table S2 Changes in water consumption with different fertilization use treatment in potato plot at Wuchuan Field Experiment Station. Water consumption values followed by the same lowercase letters for each year are not significantly different at $p < 0.05$ (e.g., 152.0a with N0P0 is not significantly different from 160.2a with N_1P_1 in 2010).

	**Treatment**	**Water consumption（mm）**		
		2008	2009	2010
	N0P0	260.3a	221.5a	152.0a
**Fertilization**	N1P1	282.6b	230.3ab	160.2a
	N2P2	316.3c	237.5bc	184.2b
	N3P3	350.5d	242.3c	202.1c
	N4P4	359.3d	265.7d	235.3d

Figure legends

Figure S**1** Average soil moisture index (SMI) trends in 0–10 cm, 10–20 cm and 20–50 cm soil across the 40 agricultural meteorological stations during 1983–2012.

Figure S2 Temporal changes in regional climate of Northern China during growing season of 1984–2007: a) air temperature (*T*); and b) precipitation (*P*).

Figure S**3** Growing season drought index trends in Northern China (NC) during 1984–2007: a) Standardized Precipitation Index (SPI); and b) Standardized Precipitation Evapotranspiration Index (SPEI). Both SPI and SPEI are for the 6-month time scale – growing season (April–September), and the trends of SPI and SPEI in every September over 1984–2007 are presented here. The black dots represent the locations of the 307 weather stations. All maps are generated via ArcMap10.2, and their projected coordinate systems are Asia North Albers Equal Area Conical.

Figure S1

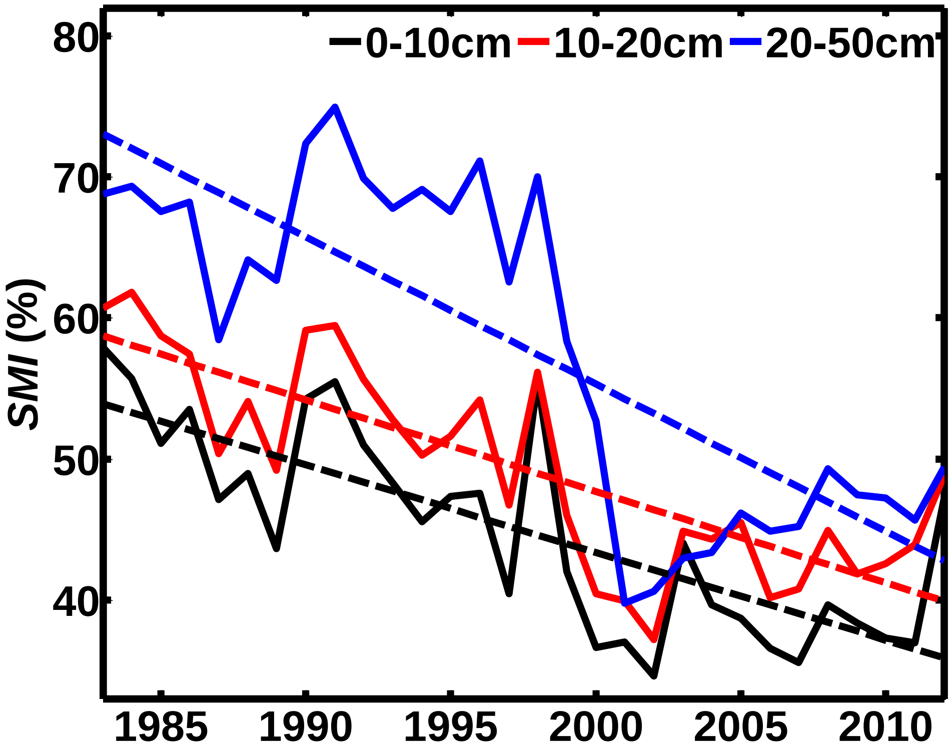

0-10cm
10-20cm
20-50cm
SMI (%)
80
70
60
50
40
1985
1990
1995
2000
2005
2010

Figure S2

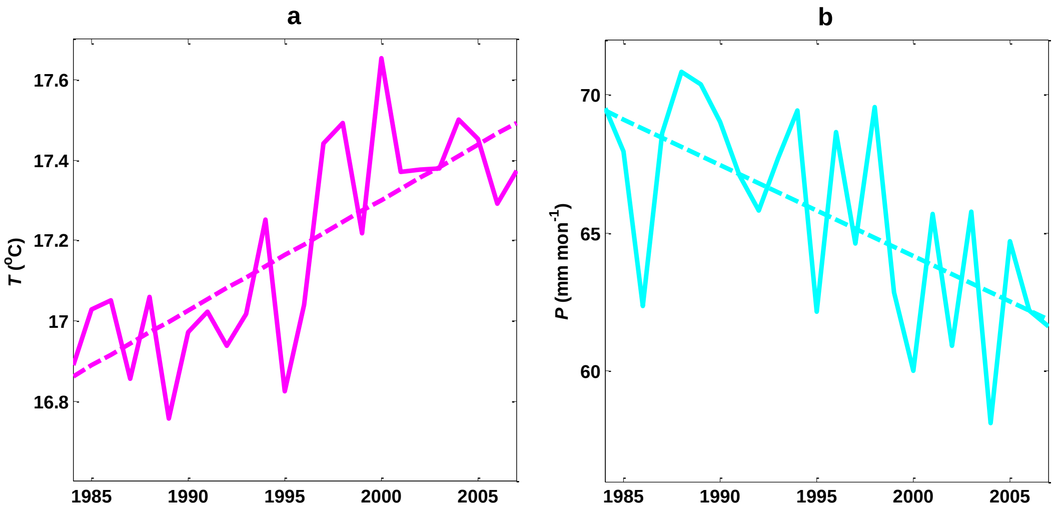
a
b
17.6
17.4
17.2
17
16.8
T (°C)
70
65
60
P (mm mon-1)
1985
1990
1995
2000
2005
1985
1990
1995
2000
2005

Figure S3

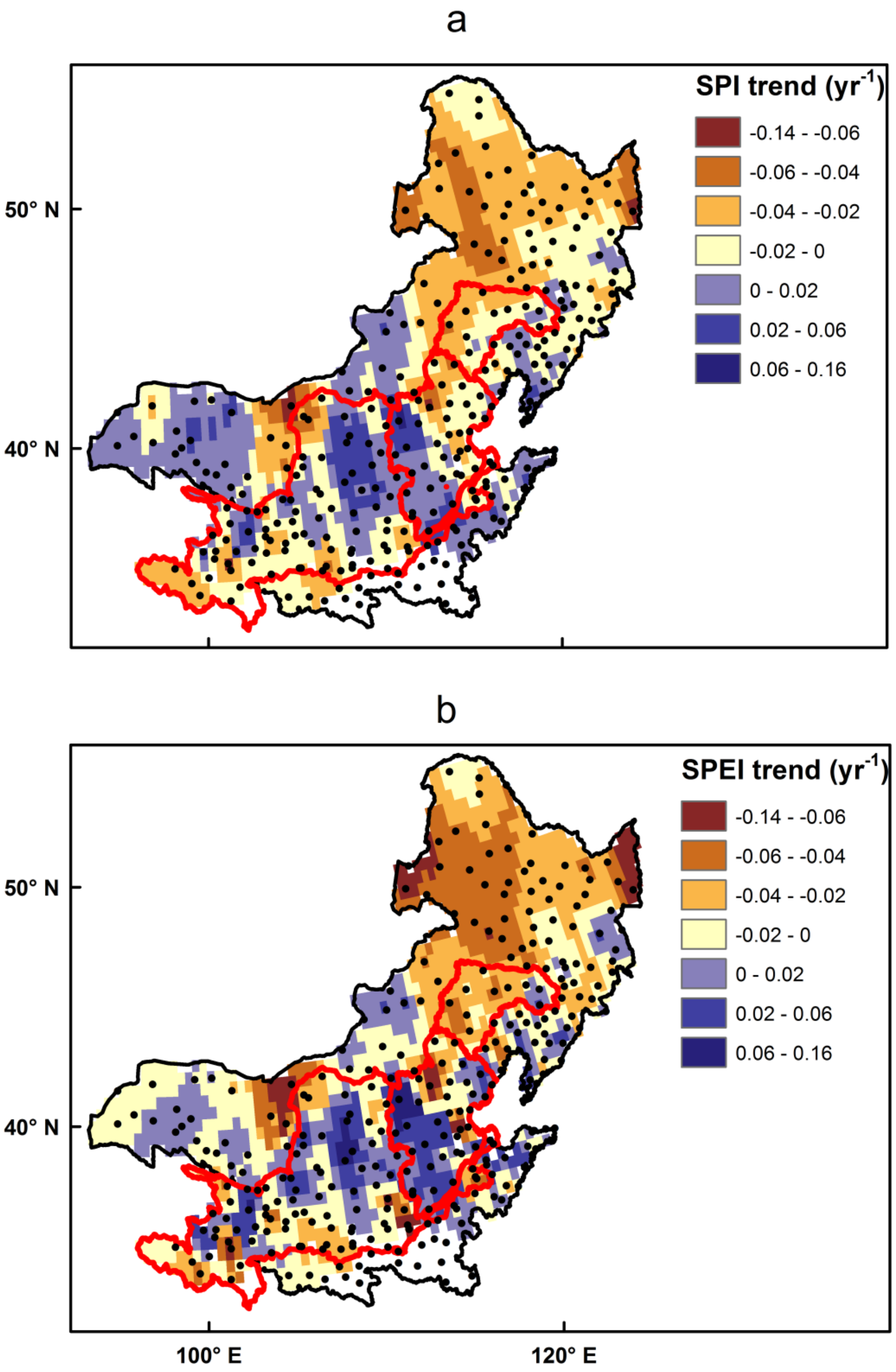
a
SPI trend (yr^{-1})
-0.14 - -0.06
-0.06 - -0.04
-0.04 - -0.02
-0.02 - 0
0 - 0.02
0.02 - 0.06
0.06 - 0.16
50° N
40° N
b
SPEI trend (yr^{-1})
-0.14 - -0.06
-0.06 - -0.04
-0.04 - -0.02
-0.02 - 0
0 - 0.02
0.02 - 0.06
0.06 - 0.16
50° N
40° N
100° E
120° E
